# Cinnamon and Derivatives as Modulators of Toll-like Receptors

**DOI:** 10.3390/ijms27188229

**Published:** 2026-09-16

**Authors:** Georges Maalouly, Marc Gemayel, Roy Khoury, Nassim Fares

**Affiliations:** 1 Laboratory of Research in Physiology and Pathophysiology, Faculty of Medicine, Saint Joseph University of Beirut, Beirut 1104 2020, Lebanon; 2 Faculty of Medicine, Saint Joseph University of Beirut, Beirut 1104 2020, Lebanon; marc.gemayel@net.usj.edu.lb (M.G.); roy.khoury5@net.usj.edu.lb (R.K.)

**Keywords:** natural compounds, immune system, toll-like receptors, cinnamon, cinnamaldehyde

## Abstract

Toll-like receptors (TLRs) are critical components of the innate immune system, acting as pattern recognition receptors and interacting with oxidative stress. TLR2, TLR4, and TLR7 play distinct yet overlapping roles in immune regulation, contributing to both physiological processes and disease progression. Therefore, modulation of TLR signaling pathways represents a promising therapeutic strategy for a wide range of diseases. Natural antioxidants have attracted considerable interest as modulators of TLRs, with cinnamon-derived phytochemicals, particularly cinnamaldehyde, emerging as promising candidates for the treatment of TLR-mediated diseases. Experimental evidence, supported by molecular docking studies, demonstrates that cinnamaldehyde directly interacts with TLR2 and TLR4. Specifically, it inhibits ligand-induced heterodimerization of TLR2 with TLR1 and TLR6, while binding to MD-2 to prevent TLR4 oligomerization and subsequent receptor activation. Furthermore, emerging evidence suggests that cinnamaldehyde may also interact with TLR7 and suppress its downstream signaling. To improve the pharmacokinetic properties, bioavailability, and targeted delivery of cinnamon-derived compounds, nanoformulations and semi-synthetic derivatives have been developed and have shown potential to modulate TLR signaling pathways. However, despite the strong biological plausibility and experimental evidence supporting the parent cinnamaldehyde scaffold as a direct TLR modulator, direct interactions between TLRs and these advanced formulations or semi-synthetic derivatives remain insufficiently documented.

## 1. Introduction

Toll-like receptors (TLRs) are critical components of the innate immune system, acting as pattern recognition receptors (PRRs) that detect pathogen-associated molecular patterns (PAMPs) and damage-associated molecular patterns (DAMPs). PAMPs are conserved molecular structures (like peptidoglycan and lipopolysaccharide) found in groups of pathogens but are absent in host cells [1], while DAMPs are endogenous molecules (like proteins, nucleic acids or metabolites) that are released or exposed by cells undergoing stress, injury, or death [2].

Several TLRs, such asTLR2, TLR4, and TLR7, are integral to both physiological and pathological processes, with distinct and overlapping roles in immune regulation, inflammation, and disease progression [3,4]. Targeting TLR signaling pathways has emerged as a promising therapeutic strategy for a wide range of diseases, including sepsis, autoimmune disorders, cancer, and malaria, with both agonists and antagonists under investigation to either dampen pathological inflammation or enhance protective immune responses [5,6,7,8]. TLRs are redox-sensitive receptors [9]. Natural antioxidants have emerged as potential modulators of TLR signaling pathways owing to their ability to target key molecules, including inhibition of LPS–TLR4 interactions, disruption of TLR2/TLR1 heterodimerization, and suppression of downstream inflammatory cascades [8,9,10,11]. Among these, cinnamon-derived compounds, particularly cinnamaldehyde, have attracted considerable attention for their anti-inflammatory properties in TLR-mediated disorders, including diabetes, inflammatory bowel disease, and periodontitis [12,13,14]. Despite these promising pharmacological activities, the clinical translation of cinnamon-derived compounds remains constrained by poor aqueous solubility, limited chemical stability, and potential toxicity [10,15,16]. These limitations have been behind the development of nanoformulations, nanoemulsions, biopolymer conjugates and semi-synthetic derivatives designed to enhance bioavailability, stability, and therapeutic efficacy [3,17] and may benefit from pharmacophore modeling [18]. However, the mechanisms by which these advanced formulations modulate TLR signaling, and whether they retain the direct receptor-targeting properties of the parent compounds, remain non-elucidated. This narrative review aims to review the role of cinnamon natural compounds, antioxidant activity mechanisms, and modern formulations and derivatives as potential modulators of TLRs.

## 2. TLRs in Physiology and Pathology

### 2.1. Subtypes and Cellular Distribution

TLRs are a family of PRRs that play a pivotal role in the innate immune system by recognizing PAMPs and DAMPs. Their discovery as a *Drosophila* fly gene involved in establishing the dorsal–ventral axis during its development, and subsequently as an antimicrobial response mediator, led to a deeper understanding of host defense and immune activation [10,11]. These receptors initiate immune responses by detecting microbial components such as lipids, nucleic acids, and proteins, as well as endogenous molecules released during tissue damage [12,13,14]. TLRs act as a bridge between innate and adaptive immunity, promoting the production of inflammatory cytokines, upregulation of co-stimulatory molecules, and functional maturation of antigen-presenting cells [14,15].

TLRs are transmembrane proteins with an extracellular domain containing leucine-rich repeats (LRRs) for ligand recognition and a cytoplasmic Toll/IL-1 receptor (TIR) domain for signal transduction. Ten types are documented in humans [10]. Ligand binding induces dimerization of TLRs, bringing their TIR domains into proximity, which is essential for downstream signaling [3,16]. TLRs are categorized into two subfamilies based on their localization: cell surface TLRs (e.g., TLR2, TLR4) and endosomal TLRs (e.g., TLR7) [3].

This distribution on the cell surface and on endosomal membranes gives TLRs the ability to recognize antigens in different cellular locations [10]. TLRs are predominantly expressed on immune cells such as macrophages, dendritic cells, and B cells, where they play a critical role in pathogen recognition and immune activation [17,19]. Additionally, TLRs are also expressed on non-immune cells, including epithelial and renal cells, where they contribute to tissue repair, inflammation, and responses to injury [20,21].

### 2.2. TLR2, 4 and 7 Ligands and Signaling Pathways

TLRs recognize microbial ligands, resulting in the activation of signaling pathways and transcription factors and inducing the expression of genes whose products are necessary for inflammatory and antiviral responses [10].

PAMPs from Gram-positive bacteria, such as peptidoglycan and bacterial lipoproteins, activate TLR2 [22]. TLR2 also responds to DAMPs released from necrotic cells or tissue damage [23]. TLR2 forms heterodimers with TLR1 or TLR6 to recognize these ligands, primarily signaling through the MyD88 pathway [16].

TLR4 recognizes lipopolysaccharides (LPSs) and signals through both MyD88 and TRIF pathways, making it multifaceted in immune responses; LPS-binding protein (LBP) and CD14 are essential for transferring LPSs to the TLR4/MD-2 complex, enabling sensitive and regulated immune responses to Gram-negative bacteria [11,16]. MD2 (myeloid differentiation protein 2) is a critical accessory protein for Toll-like receptor 4 (TLR4), playing essential roles in both the structural assembly and functional activation of the TLR4 complex, particularly in response to LPS. It is a secreted extracellular glycoprotein that binds directly to the extracellular domain of TLR4, forming the TLR4/MD2 complex necessary for LPS recognition and signal transduction [24]. MD2 contains a unique hydrophobic cavity that specifically binds the lipid A portion of LPS, facilitating the recognition of Gram-negative bacterial components [25]. MD2’s structure includes several cysteine residues forming disulfide-linked oligomers, which are important for its stability and function [26]. Upon LPS binding, the TLR4-MD2-LPS complex undergoes dimerization, which is required for downstream signaling [27]. Other ligands of TLR4 are endogenous molecules (like HMGB1, S100A8/9, heme, and fetuin-A) [28].

TLR7 is activated by single-stranded RNA (ssRNA), common in viral infections [29], and by endogenous RNA molecules released during cellular stress or damage [29].

The MyD88-dependent pathway is the primary signaling mechanism for most TLRs, including TLR4, TLR2, and TLR7. Upon ligand binding, TLRs recruit the adaptor protein MyD88, leading to the activation of transcription factors such as NFκB and AP-1. This pathway drives the production of pro-inflammatory cytokines and type I interferons, which are critical for pathogen defense and immune activation [3,12,29].

TLR4 uniquely utilizes both MyD88-dependent and MyD88-independent pathways. The TRIF-dependent pathway is activated when TLR4 is localized to endosomes, leading to the production of type I interferons via IRF3 activation. This pathway is crucial for antiviral responses and is also utilized by endosomal TLRs like TLR7 [3,12,29] (Table 1).

### 2.3. TLR in Pathology and Modulation by Cinnamon Derivative Compounds

TLR signaling dysregulation can give rise to chronic inflammation and excessive release of pro-inflammatory cytokines [32], colorectal cancer and autoimmune disorders like rheumatoid arthritis, systemic lupus erythematosus, and multiple sclerosis [33,34].

Natural compounds can act as TLR antagonists, thereby inhibiting the initiation of inflammatory cascades triggered by PAMPs or DAMPs. By interfering with TLR-mediated signaling, these compounds reduce the production of pro-inflammatory cytokines and chemokines, which are central to the development and progression of autoimmune and inflammatory diseases [35,36].

Among these compounds, cinnamon, a widely used spice arising from the bark of trees in the *Cinnamomum* genus (family Lauraceae), is known for an extensive history in culinary and medicinal applications. The two most common species are *Cinnamomum zeylanicum* (Ceylon cinnamon) and *Cinnamomum cassia* (cassia cinnamon) [37,38].

Cinnamon contains a variety of natural derivatives and bioactive compounds responsible for its health benefits and flavor. Cinnamaldehyde is a major aldehyde component of cinnamon essential oil, responsible for its characteristic flavor and aroma. It exhibits antioxidant, anti-inflammatory, antimicrobial, and antidiabetic properties [39]. Cinnamic acid, a phenolic acid, is another major derivative with antioxidant and therapeutic effects [40]. Cinnamate derivatives include various esters and salts of cinnamic acid that contribute to biological activities [41]. Cinnamyl alcohol is an aroma compound and participates in the bioactivity of cinnamon, while cinnamyl acetate, an ester, is an aroma compound and contributes to health effects [42]. The essential oil of cinnamon is a complex mixture of volatile secondary metabolites, with trans-cinnamaldehyde being the major component. Other volatiles include eugenol and various terpenoids [43]. Additionally, cinnamon contains a variety of phenolic compounds, including tannins and flavonoids, which contribute to its antioxidant properties [44] (Table 2).

Additional benefits of cinnamon comprise antidiabetic effects with improvement of insulin sensitivity and blood glucose regulation, documented with cinnamaldehyde and MHCP (Methyl-hydroxy Chalcone Polymer) [42], and antimicrobial outcomes due to eugenol and cinnamaldehyde [38,45]. Finally, coumarin is a naturally occurring compound found in cinnamon, known for its sweet, hay-like aroma, and it is used as a flavoring agent in foods and fragrances. Cassia cinnamon contains high levels of coumarin, up to 1% by weight, while Ceylon cinnamon contains very low levels of coumarin, typically less than 0.004% [46]. Coumarin in cinnamon exhibits anticoagulant, antithrombotic, antimicrobial, antioxidant, and potential anticancer properties [47]. However, coumarin can cause liver toxicity in some individuals and experimental animals; due to its potential toxicity, especially in Cassia cinnamon, regulatory agencies (e.g., European Food Safety Authority) have set tolerable daily intake (TDI) limits (e.g., 0.07–0.1 mg/kg body weight/day) [48].

Cinnamon exhibits anti-inflammatory and immunomodulatory effects that may benefit TLR-mediated diseases, including autoimmune, metabolic, and neuroinflammatory disorders [49,50]. Evidence is strongest in preclinical models, with clinical data in autoimmune conditions [51,52] (Table 3).

#### 2.3.1. Modulation of Endotoxin and Infection-Induced Inflammation

In a mouse model of acute lung injury induced by lipopolysaccharide (LPS), *Cinnamomum cassia* bark essential oil was able to suppress the TLR4/MyD88/NF-κB signaling pathway [53]. Moreover, another study suggested that cinnamaldehyde can suppress both TLR4 and NOX4 activation during endotoxemia and inhibited cardiac dysfunction, cardiac inflammatory infiltration and the levels of TNF-α, IL-1β and IL-6 in LPS-stimulated rats by blocking TLR4, NOX4, MAPK and autophagy signaling [54]. In mice induced by LPS, the anti-inflammatory effect of cinnamaldehyde was mainly related to the inhibition of TLR4/MD2, MyD88, and NLRP3 [55]. Furthermore, in Aggregatibacter actinomycetemcomitans-infected THP-1 cells, pretreatment with trans-cinnamic aldehyde significantly inhibited infection-induced expression of the TLR signaling pathway [56].

Cinnamon was also suggested as a promising modulator of the hyperinflammatory state in COVID-19 infection, although data are scarce and mostly experimental [57]. One study comparing cinnamon compounds to dexamethasone in THP-1 monocytes found that cinnamaldehyde interferes with the dimerization of TLR4 and suggested that supplementary treatment with Ceylon cinnamon may allow administration of lower doses of dexamethasone and may modulate angiogenesis in COVID-19 [58].

#### 2.3.2. Modulation of Synovial Inflammation, Osteoarthritis and Rheumatoid Arthritis

Cinnamon and its active compound, cinnamaldehyde, are used in traditional medicine for osteoarthritis and have demonstrated anti-inflammatory effects in osteoarthritis models, particularly in reducing synovial inflammation. One study focusing on human fibroblast-like synoviocytes to investigate the effects of cinnamaldehyde on synovial inflammation confirmed inhibition of inflammation in LPS-induced human fibroblast-like synoviocytes via blocking the TLR4/MyD88 signaling pathway [59]. A previous study has demonstrated that cinnamaldehyde significantly suppressed LPS-stimulated NF-κB activation in chondrocytes [60].

On the other hand, a randomized double-blind clinical trial confirmed that cinnamon supplementation can be an interesting adjunct treatment to ameliorate inflammation and clinical symptoms in patients with rheumatoid arthritis [52]. Additionally, Baihu-Guizhi decoction (BHGZD), a traditional Chinese medicine containing cinnamic acid and other natural compounds, may have anti-rheumatic properties; an interaction network demonstrated that cinnamic acid targets TLR4/PI3K/AKT/NFκB/NLRP3 signaling-mediated pyroptosis in rheumatoid arthritis [61]. Cinnamic acid in this decoction effectively decreased the disease severity of the adjuvant-induced arthritis-modified rat model by inhibiting TLR4/PI3K/AKT signaling [61].

#### 2.3.3. Modulation of Colitis Models

In acetic acid-induced colitis in rats, cinnamic acid effectively reduced inflammation by inhibiting the activities of TNF-α, IL-6, and myeloperoxidase and downregulating the expression of TLR4 [62]. Moreover, cinnamaldehyde ameliorated the symptoms of dextran sulfate sodium-induced colitis by decreasing disease activity, relieving colonic pathology and decreasing systemic inflammation by reducing serum myeloperoxidase, TNF-α, IL-6, and IL-1β, and downregulating the TLR4/NF-κB signaling pathway [63].

#### 2.3.4. Modulation of Lupus

In TLR7 agonist-induced lupus in mice, cinnamon effectively protected the gut–liver axis and normalized the enhanced expression of TLR7, p-NFκB/NFκB, SOD1 and SOD2 in liver induced by lupus [64]. In another study using the same model, cinnamon was able to dampen TLR7-MYD88 pathway activation in parallel to antioxidant and antiapoptotic neuroprotective effects in hippocampal cells [65].

#### 2.3.5. Modulation of Neuroinflammation

Robust studies have shown that the TLR4/NF-*κ*B signaling pathway plays a key role in the pathogenesis of neuroinflammation [66]. In a mouse model of permanent cerebral ischemia, cinnamaldehyde reduced the neurological deficit scores, brain edema and infarct volume and suppressed the activation of signal transduction molecules, including TLR4 [67]. Furthermore, in a global cerebral ischemia model, cinnamaldehyde reduced synaptic protein overexpression, decreased pro-inflammatory cytokine (IL-6, IL-1β, TNF-α) levels, and suppressed TLR4/MyD88/MAPKs pathway activation in GCI rats or primary microglia induced by LPS [68]. Additionally, in an experimental model of Alzheimer disease (aluminum chloride-induced cognitive impairment) in zebrafish, cinnamaldehyde mitigated neuroinflammation and offered neuroprotective benefits by suppressing the TLR4/NF-κB pathway [69].

#### 2.3.6. Modulation of Metabolic Syndrome and Diabetes

Fructose consumption induces metabolic syndrome and increases cardiovascular risk. Fructose-fed rats displayed metabolic syndrome with elevated serum ox-LDL, cardiac oxidative stress, inflammation and fibrosis, with an increase in scavenger receptor CD36, TLR4, TLR6, and IL-1R-associated kinase 4/1 (IRAK4/1) signaling in animals and H9c2 cell models [70]. In this model of metabolic syndrome, cinnamaldehyde reduced cardiac oxidative stress, NLRP3 inflammasome activation and TGF-β/Smads signaling by inhibiting CD36-mediated TLR4/6-IRAK4/1 signaling under fructose induction [70]. In experimental diabetic rat kidneys, cinnamon reversed an increase in mast cell and TLR4 expression in rats with diabetes [71].

#### 2.3.7. Modulation of Cancer

In contrast to hyperinflammatory states where cinnamon compounds contribute to downregulation of TLR pathways, the context of tumor microenvironments may present a different perspective: while cancer cells may often hijack or dysregulate signaling pathways (including TLR4 axes) to foster immunosuppression, cinnamaldehyde in these models interacts with TLR4 to normalize their behavior [72]. In one study on prostate cancer-associated fibroblasts, cinnamaldehyde was able to reverse the immunosuppressive function of these fibroblasts by activating the TLR4 pathway in these cells [72]. Similar findings were retrieved in another study where cinnamaldehyde caused apoptosis of myeloid-derived suppressor cells through the activation of TLR4 [73].

**Table 3:** Selected TLR-mediated pathologies modulated by cinnamon compounds.

TLR-Mediated Pathology	Relevant TLR	Cinnamon Compound(s)	Reported Effect	Selected References
Osteoarthritis synovial inflammation	TLR4, with interaction also suggested for TLR2	Cinnamaldehyde	Anti-inflammatory; inhibits TLR4/MyD88 signaling	[59]
Rheumatoid arthritis	TLR2, TLR4	Cinnamon supplementation; likely cinnamaldehyde-rich preparations	Improved DAS-28, pain, swollen/tender joints, CRP, TNF-α in a clinical study	[52]
Endotoxin-induced systemic inflammation	TLR4	Cinnamaldehyde, linalool	Lowers pro-inflammatory cytokines and suppresses TLR4/MyD88/MD2 signaling	[55]
COVID-19-related hyperinflammation	TLR4	Cinnamaldehyde; cinnamon extract	Mitigates inflammatory signaling and interferes with TLR4 dimerization	[58]
Lupus/neuropsychiatric lupus erythematosus	TLR7, with broader innate immune modulation	Cinnamomum cassia; cinnamaldehyde	Alleviates disease features in a TLR7 agonist-induced lupus model	[51]
Colitis models	TLR4	Cinnamic acid	Ameliorates colitis and inhibits TLR4 pathway	[74]
Neuroinflammation	TLR4	Cinnamaldehyde	Ameliorates ischemia and cognition and mitigates TLR4 pathway activation	[67,68]
Metabolic syndrome and diabetes	TLR4	Cinnamaldehyde	Decrease mast cells and TLR4 in rat kidneys Decrease cardiac fibrosis and inflammation	[70,71]
Prostate cancer/tumor-related inflammation	TLR4, plus NF-κB, JAK/STAT3, AKT, Nrf2/Keap1-ARE	Cinnamaldehyde	Anti-proliferative, pro-apoptotic, anti-angiogenic, anti-inflammatory effects	[75]

## 3. Pharmacokinetics, Hepatic Metabolism, and Bioavailability of Cinnamon

Cinnamaldehyde is a major constituent of cinnamon and contributes to its biological properties. The pharmacokinetics of cinnamaldehyde are determined by its chemical structure, which includes a reactive α,β-unsaturated aldehyde moiety, and it is associated with poor aqueous solubility and rapid first-pass metabolism, complicating its efficacy as a therapeutic agent [76].

Bioavailability of cinnamaldehyde was documented to be 100% in both fasted and fed-state gastric and intestinal fluids in one study [77]. Cinnamaldehyde is rapidly absorbed after oral administration. It is metabolized extensively in the stomach, small intestine, and liver before entering systemic circulation [78].

A pharmacokinetic study of oral and intravenous administration of cinnamaldehyde in rats found a double-peak phenomenon after oral intake but not after intravenous injections, suggesting an enterohepatic circulation of cinnamaldehyde [79]. Similar findings were also reported for 2-hydroxycinnamic acid in another study after cinnamon consumption in overweight adults [80]. Cinnamon metabolites may appear in plasma as early as 0.5 h after consumption, highlighting the rapid absorption of these compounds [80]. Pharmacokinetic analysis in rats revealed that cinnamaldehyde has a relatively long half-life of approximately 6.7 h, indicating sustained presence in the plasma after administration [79].

Cinnamaldehyde is rapidly metabolized into several compounds, including cinnamic acid, cinnamyl alcohol, and methyl cinnamate. These metabolites are detectable in plasma and are interconvertible, with cinnamaldehyde being detoxified into cinnamic acid via aldehyde dehydrogenase (ALDH) and alcohol dehydrogenase (ADH) [79,81,82].

Upon incubation with human liver microsomes and human liver S-9 fraction, cinnamaldehyde is rapidly oxidized into cinnamic acid [77]. Cinnamic acid appears to be the main metabolite of *Cinnamomum zeylanicum* in human plasma after oral administration of 5g, with maximal plasma concentration (Cmax) of 1.9 ± 1.5 μmol/L, a time to reach maximal concentration (Tmax) of 15 min, and an elimination half-life (T½) of 36 min [83]. Cinnamic acid is β-oxidized to benzoate in the liver as sodium salt (NaB) or benzoyl-CoA [84].

In addition to these compounds, coumarin and hydroxycoumarin glucuronide significantly increase in human plasma after ingestion of a meal containing cinnamon. Coumarin absorption from cinnamon tea is fast, leading to the highest peak concentrations of 7-hydroxycoumarin, suggesting a potentially higher risk compared to other cinnamon formulations [85].

Cinnamon bark contains water-soluble polyphenolic polymers, including procyanidins (type A proanthocyanidins) and catechins. Polyphenols from cinnamon undergo limited absorption in the small intestine but are metabolized by colonic bacteria into smaller phenolic compounds, some of which may be biologically active [86].

Key limitations in therapeutic use of cinnamon in humans are related to pharmacokinetic challenges and variability in bioactive compounds. For example, Cassia cinnamon may contain up to 1% coumarin, a compound with potential hepatotoxicity, while Ceylon cinnamon contains much less (<0.004%) [46]. Poor solubility and instability, and incomplete information on the absorption, distribution, metabolism, and excretion of cinnamon’s active compounds in humans, challenge optimal dosing and interaction prediction [87,88].

## 4. Antioxidant Activity of Cinnamon and Modulation of TLR

### 4.1. Antioxidant Activity of Cinnamon

Cinnamon exhibits significant antioxidant activity related to its bioactive compounds. The mechanisms by which cinnamon acts as an antioxidant are multifaceted and involve several chemical and biological pathways. Comparative studies reveal that cinnamon’s antioxidant activity is comparable to or higher than some synthetic antioxidants used in food preservation [89].

Polyphenols, flavonoids, cinnamic acid derivatives, and coumarins have direct free radical scavenging capacity, documented in vitro by the capacity to reduce DPPH• (2,2-diphenyl-1-picrylhydrazy), ABTS+ (2,2’-azino-bis(3-ethylbenzothiazoline-6-sulfonic acid)) and hydroxyl radicals [89]. Fractionation studies show that water and ethanol extracts, rich in phenolics and flavonoids, have the highest antioxidant activities [90].

Additionally, essential oils from cinnamon, particularly those rich in eugenol, also contribute to free radical scavenging [91].

On the other hand, cinnamon extracts demonstrate metal-chelating activity, preventing the catalysis of free radical formation by transition metals (e.g., iron) and further reducing oxidative damage [45]. The reducing power of cinnamon extracts is related to electron donation to unstable molecules, stabilizing them and preventing oxidative chain reactions [92]. Furthermore, cinnamon extracts inhibit lipid peroxidation, as demonstrated in β-carotene-linoleate assays, protecting cell membranes from oxidative damage [93].

Enhancement of endogenous antioxidant enzymes is another facet of the antioxidant activity of cinnamon. Cinnamon compounds have been shown to modulate the activity of endogenous antioxidant enzymes, such as superoxide dismutase (SOD), catalase, and glutathione peroxidase, and restore the balance of antioxidant defenses in conditions like diabetes and metabolic syndrome [94], as also suggested by animal models of diseases [95] (Table 4).

### 4.2. Structure–Reactivity Analysis of Cinnamon Antioxidant Activity

The antioxidant efficacy of *Cinnamomum* species is predominantly governed by a structurally diverse profile of phenylpropanoids and polyphenols, whose mechanistic reactivities can be elucidated through quantitative structure–activity relationship (QSAR) paradigms [96]. Key contributors include eugenol (4-allyl-2-methoxyphenol), cinnamaldehyde, cinnamic acid derivatives, and oligomeric proanthocyanidins. Phenolic constituents such as eugenol act primarily as chain-breaking antioxidants via hydrogen atom transfer (HAT) and single electron transfer–proton transfer (SET-PT) mechanisms, where radical scavenging potency correlates inversely with phenolic O–H bond dissociation energy (BDE) and directly with resonance stabilization afforded by electron-donating substituents like the ortho-methoxy group in the guaiacol core [97]. Conversely, cinnamaldehyde features an αβ-unsaturated carbonyl moiety that displays modest direct radical scavenging in abiotic assays yet functions as a potent electrophilic Michael acceptor capable of modulating cellular redox homeostasis [98]. Specifically, cinnamaldehyde covalently modifies nucleophilic cysteine thiols within the Kelch-like ECH-associated protein 1 (Keap1), promoting the nuclear translocation of nuclear factor erythroid 2-related factor 2 (Nrf2) and upregulating downstream antioxidant response element (ARE)-driven phase II detoxifying enzymes [98].

Derivatives of cinnamaldehyde and cinnamic acid with hydroxy or methoxy groups, especially at specific positions on the aromatic ring, show enhanced antioxidant activity. For example, 4-hydroxy-3,5-di-tert-butylcinnamic acid exhibits high antioxidant activity, as predicted by quantum-chemical calculations and confirmed experimentally [99]. Similarly, hydroxy-bearing cinnamaldehyde compounds demonstrate potent DPPH radical-scavenging activity [100]. Moreover, among cinnamic acid derivatives, compounds containing catechol or o-methoxy groups on the aromatic ring display strong antioxidant effects, suggesting that electron-donating substituents enhance radical-scavenging ability [101].

Quantitative structure–activity relationship (QSAR) and computational analysis studies suggest that lower O-H bond dissociation energies in hydroxycinnamic acids correlate with higher antioxidant activity [102]. Additionally, calculated indices of reactivity with respect to active oxygen species support the experimental findings that certain hydroxy- and methoxy-derivatives are more effective antioxidants [99].

Finally, the antioxidant activity of proanthocyanidins in cinnamon is strongly dependent on their degree of polymerization, type of interflavan linkage, galloylation, and structural modifications such as condensation with aldehydes, which are influenced by both natural composition and processing [103,104].

These integrated electronic and biological mechanisms account for extract-dependent variations and provide a robust molecular framework for designing targeted antioxidant therapeutics (Table 5).

### 4.3. Modulation of TLR2 and TLR4

Molecular docking simulation shows that cinnamaldehyde can bind to threonine 136 on TLR4 and histidine 318 on TLR2 with binding energy of −5.12 kcal/mol and −4.04 kcal/mol, respectively [59]. Mechanistic studies revealed that cinnamaldehyde suppresses the activation of NF-kB and IRF3 induced by lipopolysaccharide, a TLR4 agonist, but is not able to inhibit the activation of NF-kB or IRF3 induced by MyD88-dependent (MyD88, IKKbeta) or TRIF-dependent (TRIF, TBK1) downstream signaling components. However, oligomerization of TLR4 induced by lipopolysaccharide is suppressed by cinnamaldehyde, with downregulation of NF-kB activation. These studies demonstrated that one of the main molecular targets of cinnamaldehyde in TLR4 signaling is the oligomerization process of the receptor [105]. Additionally, cinnamaldehyde inhibits TLR4 oligomerization induced by LPS in co-immunoprecipitation experiments. The observation that the saturated analog of cinnamaldehyde, 2-phenylpropionaldehyde, was unable to effect this inhibition, as well as the reactivity of cinnamaldehyde with other cysteine-exposing proteins, suggests the formation of a covalent adduct, cinnamaldehyde/MD-2 [106]. However, this was not documented in another study testing whole cinnamon extract [107]. When trans-cinnamaldehyde is tested in the HEK-TLR2/HEK-TLR4 comparative assay system for TLR4-specific antagonism, the significant decrease in the inflammatory signal in HEK-TLR2 and HEK-TLR4 cells indicates an involvement of trans-cinnamaldehyde in both TLR2 and TLR4 signaling pathways, indicating that its mode of action goes beyond sole TLR4 antagonism and the reported suppression of TLR4 receptor dimerization [107]. In this study, synergistic anti-inflammatory effects were observed for combinations of trans-cinnamaldehyde with *p*-cymene, cinnamyl alcohol or cinnamic acid on downstream pathways (IL-8, Akt, and IκBα) [107].

The physical mechanism of this blockade of TLR4 is driven by the highly electrophilic αβ-unsaturated carbonyl moiety of cinnamaldehyde [108]. This structure acts as a Michael acceptor, forming covalent adducts via Michael addition with nucleophilic, solvent-accessible cysteine residues on target proteins [108]. The accessory protein MD-2 contains a central, deep hydrophobic pocket that accommodates five of the six lipid A acyl chains of LPS, while the sixth acyl chain remains exposed to bridge to a secondary TLR4 monomer and promote homodimerization [106] (Table 4).

Covalent modification of Cys133 by cinnamaldehyde blocks the entry of lipid A chains or destabilizes the electrostatic potentials at the dimerization interface [109]. By modifying these cysteine residues, cinnamaldehyde prevents the conformational changes and charge-dependent ionic interactions required to bring the intracellular TIR domains of the two TLR4 monomers into proximity [108]. In comparison to other MD2 inhibitors that compete directly with full-length MD-2 for the TLR4 ectodomain [110], cinnamaldehyde achieves its antagonistic profile by covalently modifying the native pocket of MD-2 to disrupt homodimerization and subsequent adapter recruitment [108]. The inhibition of LPS-induced NF-kB activation by cinnamaldehyde was reversed by thiol donors in Ba/F3 cells expressing TLR4 in one study, suggesting that the sulfhydryl-modifying activity of cinnamaldehyde (decreasing intracellular thiol levels, possibly due to the α,β-unsaturated carbonyl moiety) is correlated with the suppressive effects on TLR4 signaling and oligomerization [105].

Similarly, cinnamaldehyde attenuates TLR2-dependent inflammatory signaling. In stimulated human monocyte (THP-1) and HEK-reporter models, cinnamaldehyde reduces Pam2CSK4-induced TLR2 activation [107]. While TLR2 undergoes heterodimerization with TLR1 or TLR6 upon lipid ligand binding, cinnamaldehyde may intercept this cascade at the membrane level, restricting the phosphorylation of Akt and p38 and downstream nuclear translocation of NF-κB [107,111] (Table 6).

### 4.4. Modulation of TLR7

Emerging data suggests that cinnamon may modulate TLR7 expression and activity. In imiquimod-induced murine lupus, cinnamon supplementation significantly decreased the expression of TLR7 and p-NFκB/NFκB in the liver tissue of lupus mice [64]. In another study using the same lupus model, hippocampal expression of MyD88 protein, a key adaptor in the TLR7 signaling pathway, was significantly enhanced in lupus mice compared to dampened in cinnamon-treated lupus mice [65]. Additionally, a molecular docking study revealed that cinnamaldehyde interacts with TLR7 at multiple sites: when the TLR7/imiquimod complex was docked with cinnamaldehyde, the latter seemed to lodge in the same pocket of the TLR7 homodimer, interacting with ASN215 via a hydrogen bond, while three other non-covalent interactions were also present at proline 261 (PRO261), CYS270, and TYR468 [51]. A cellular thermal shift assay supported the interaction between cinnamaldehyde and TLR7 [51] (Table 4).

### 4.5. Indirect Modulation of TLR Signaling by Cinnamon Antioxidant Activity

The relationship between oxidative stress and TLRs seems to be bidirectional. TLRs are redox-sensitive receptors; conversely, TLR activation can promote oxidative stress, creating a feedback loop that amplifies inflammation and tissue damage [9,112].

Cinnamon antioxidants reduce ROS, malondialdehyde (MDA), and nitric oxide (NO), upregulate SOD and catalase, and modulate redox-sensitive transcription factors, such as NF-κB downstream of TLR activation [113,114,115]. ROS reduction prevents NF-κB nuclear translocation and pro-inflammatory gene expression [114,116]. Upregulation of SOD and catalase attenuates TLR4-mediated activation of NF-κB and NLRP3 inflammasome pathways [115,117].

In addition, cinnamon supplementation decreases systemic inflammatory biomarkers (IL-6, CRP, TNF-α) and increases total antioxidant capacity, indirectly affecting TLR signaling [118,119,120]. Cinnamaldehyde activates Nrf2, upregulates antioxidant enzymes, and protects against oxidative stress-induced endothelial dysfunction [121,122]. In hepatic injury and lung ischemia-reperfusion models, Nrf2 deficiency leads to increased TLR4-driven inflammation and tissue damage, indicating that Nrf2 regulates TLR-mediated inflammatory responses [123,124].

Cinnamon extracts also have immunomodulatory effects on antigen-presenting cells (APCs) and T cells. They promote regulatory APCs and IL-10-producing T cells, suppress pro-inflammatory cytokines, and modulate immune responses relevant to TLR-mediated inflammation [125,126]. Procyanidin oligomers inhibit splenocyte proliferation and cytokine production, supporting immune regulation [127].

In addition, cinnamon antioxidants modulate kinases such as PI3K/Akt and NIK/IKK, preventing oxidative stress-induced phosphorylation changes in adaptor proteins downstream of TLR activation [128].

## 5. Nanoformulations and Semi-Synthetic Derivatives of Cinnamon: Effect on TLR

### 5.1. Nanoformulations

Cinnamon oil and cinnamaldehyde suffer from poor water solubility and structural instability, which severely restrict their clinical utility in targeting systemic inflammation [129]. Formulating the volatile oils into cinnamaldehyde-based self-nanoemulsions protects the bioactive compounds from environmental degradation while retaining their intrinsic ability to inhibit inflammatory markers; this was demonstrated in particular for Neutrophil-Activating Protein 3 (NAP3 or CXCL1) inhibition [129]. CXCL1 is a downstream inflammatory gene induced by TLR signaling, especially via TLR4/TLR2 activation through NFκB and related pathways [130]. While direct evidence that nanoemulsion formulation of cinnamaldehyde retains a direct modulatory effect on TLR is lacking, these data support this hypothesis. By maintaining high local bioavailability at the site of injury or disease, these nano-systems may ensure prolonged, controlled release, theoretically capable of dampening overactive TLR4 pathways.

Green-synthesized Cinnamon Silver Nanoparticles (AgNPs) represent a unique biogenic platform. In these nanostructures, the bioactive components of cinnamon extract (principally cinnamaldehyde and associated polyphenols) act as both the reducing and capping agents for the metallic silver core [131]. Silver nanoparticles induce apoptosis through the toll-like receptor 2 pathway in cultured cells [132]. However, in vivo models evaluating biogenic cinnamon–silver combinations show a robust, dose-dependent decrease in systemic pro-inflammatory biomarkers (TNF-α, IL-6) and a concurrent optimization of mucosal and systemic immune responses, suggesting a successful downregulation of chronic hyperinflammatory TLR signaling [132]. The direct effect of this formulation on TLR needs future confirmation.

Similarly, gold nanoparticles from *Cinnamomum verum* demonstrated an inhibitory effect on TLR/NF-κB signaling in murine-induced Parkinson disease [132].

Furthermore, cinnamic acid nanoparticles led to a notable decrease in inflammatory cytokine levels and mitigated the induction of TLR4 and MyD88 levels in an acute hepatitis model in rats [133]. Additionally, in docking analysis, one symmetric synthetic cinnamic derivative (6h) was shown to fit well inside the structure of MD2: one alkoxy group of symmetrical ester bonds forms a key double hydrogen bond with the residues SER-392 and SER-394, while another symmetrical carbonyl oxygen interacts with ASN-339 via an additional H-bond, further reinforcing the binding of ligands [134].

### 5.2. Semi-Synthetic Derivatives

The development of semi-synthetic derivatives of cinnamaldehyde focuses on optimizing its anti-inflammatory, antimicrobial, and immunomodulatory profiles while addressing its dose-dependent cytotoxicity.

4-Hydroxycinnamaldehyde-Galactosamine (HCAG) is a semi-synthetic conjugate designed specifically to widen the therapeutic window. Conjugation with galactosamine yields a 40-fold reduction in cytotoxicity while fully preserving its anti-inflammatory footprint [135]. It was demonstrated to decrease TLR4 gene expression and NFκB activation, but a direct effect on TLR4 modulation is lacking [135]. Similarly, a DM1 cinnamoyl derivative was demonstrated to counter LPS-induced TLR4 overexpression [136]. However, direct interaction with TLR4 oligomerization was not studied. On the other hand, α-bromo-4-chlorocinnamaldehyde reduced TLR4 expression in cardiac tissue in CVB3-induced viral myocarditis without proven ability to interact with TLR4 [137].

Furthermore, one of the 9-Cinnamyl-9*H*-purine derivatives (5e), synthetized by alterations to the hydroxyl group in the cinnamyl group, was able to disrupt the TLR4–MyD88 protein interaction, leading to the suppression of NFκB signaling pathway suppression [138]. Another compound (2i), synthetized from a series of cinnamamides, was found to block LPS-induced MD2/TLR4 pro-inflammatory signaling activation in vitro and to attenuate LPS-caused sepsis by directly targeting and binding MD2 in Arg90 and Tyr102 residues and inhibiting MD2/TLR4 complex formation [139].

In contrast, the vast majority of semi-synthetic cinnamaldehyde derivatives documented in the medicinal chemistry literature—most notably structured series of ethers, sulfides, sulfones, Schiff bases, and dimeric analogs—have been evaluated predominantly for their antibacterial or cytotoxic/anticancer efficacy [140,141]. Consequently, there remains a paucity of direct experimental evidence demonstrating their regulatory effects on Toll-like receptor (TLR) platforms. Because native cinnamaldehyde’s well-established inhibition of TLR4 oligomerization relies heavily on the spatial and electrophilic integrity of its alpha-beta-unsaturated carbonyl pharmacophore, modifying these structural attributes often shifts the pharmacological properties toward non-specific cellular toxicity or antimicrobial targets. Thus, while the parent cinnamaldehyde scaffold displays robust biological plausibility as a TLR-active lead compound, the definitive TLR-modulating fingerprints of its more complex semi-synthetic derivatives remain largely unconfirmed [141,142].

## 6. Conclusions

Cinnamon natural compounds show ability to modulate TLRs, especially TLR2, 4 and 7. Nanoformulations and semi-synthetic derivatives, developed to optimize pharmacokinetics and delivery of cinnamon compounds, have emerged as potential modulators of TLR signaling pathways, but data on direct interaction with TLR2, 4 and 7 remains to be fully explored. Future studies should focus on detailing mechanistic and kinetic characteristics of these modern formulations of cinnamon to translate into therapeutics of TLR-related diseases.

## Figures and Tables

**Table 1:** Comparison table of signaling cascades of TLR2, 4 and 7 [30,31].

Receptor	Location	Main Ligands	Adaptor Proteins	Key Pathways Activated	Main Cytokines Produced
TLR2	Cell membrane	Peptidoglycan, etc.	MyD88	NF-κB, MAPK	Pro-inflammatory cytokines
TLR4	Cell membrane	LPS, DAMPs	MyD88, TRIF	NF-κB, MAPK, IRF3/7	Pro-inflammatory, Type I IFNs
TLR7	Endosome	ssRNA	MyD88	NF-κB, IRF7	Type I IFNs, inflammatory cytokines

**Table 2:** Key natural compounds and derivatives of cinnamon.

Compound Name	Description & Properties
Cinnamaldehyde	The major component of cinnamon essential oil; responsible for its characteristic flavor and aroma. Exhibits antioxidant, anti-inflammatory, antimicrobial, and antidiabetic properties.
Cinnamic Acid	Another major derivative with antioxidant and therapeutic effects.
Cinnamate Derivatives	Includes various esters and salts of cinnamic acid, contributing to biological activities.
Cinnamyl Alcohol	Present in cinnamon oil, with reported biological activity.
Cinnamyl Acetate	Contributes to aroma and potential health effects.
Eugenol	A phenolic compound with antimicrobial and antioxidant properties.
Coumarin	Present in some cinnamon species, known for anti-inflammatory effects.
Polyphenols & Tannins	Includes a range of antioxidant compounds contributing to health benefits.
Methyl-hydroxy Chalcone Polymer (MHCP)	A polymeric compound with antidiabetic potential.

**Table 4:** Key mechanisms of cinnamon antioxidant activity.

Mechanism of Antioxidant Activity of Cinnamon	Description
Free Radical Scavenging	Cinnamon polyphenols and flavonoids can directly scavenge free radicals such as DPPH•, ABTS+, and OH−, reducing oxidative stress.
Metal Chelation	Cinnamon extracts can chelate metal ions, which prevents metal-catalyzed formation of reactive oxygen species (ROS).
Reducing Power	Cinnamon demonstrates the ability to donate electrons, thereby neutralizing free radicals and reducing oxidative damage.
Enhancement of Endogenous Antioxidants	Cinnamon may help restore or enhance the activity of endogenous antioxidant enzymes, balancing oxidative status in disease models.

**Table 5:** Cinnamon key compounds and their structure–activity relationships.

Compound/Class	Structural Features	Antioxidant Activity & Reactivity
Eugenol	Phenolic ring with allyl group	High antioxidant activity; activity correlates with phenolic content and structure
Cinnamaldehyde	α,β-unsaturated aldehyde	Exhibits antioxidant, anti-inflammatory, and enzyme inhibitory activities; derivatives show variable activity based on modifications
Cinnamic Acid Derivatives	Phenylpropanoid backbone, carboxylic acid group	Antioxidant activity linked to π-electron systems and O-H bond dissociation energies; hydroxy-derivatives show enhanced activity
Proanthocyanidins	Oligomeric flavonoids, multiple phenolic groups	Contribute to total phenolic content and overall antioxidant capacity of cinnamon extracts

**Table 6:** Mechanistic insights into cinnamaldehyde interaction with TLR2, 4 and 7.

TLR Modulated by Cinnamaldehyde	Mechanistic Interactions Between Cinnamaldehyde and TLR Pathway	Selected References
TLR2	Binding to histidine 318 on TLR2	[59]
	Possible inhibition of heterodimerization of TLR2 with TLR1 or TLR6	[107,111]
TLR4	Binding to threonine 136 on TLR4 Formation of a covalent adduct, cinnamaldehyde/MD-2 (interaction with cysteine Cys133 residue on MD2); sulfhydryl-modifying activity of cinnamaldehyde Inhibition of TLR4 oligomerization	[59,105,106]
TLR7	Cinnamaldehyde interacts with TLR7 on multiple sites in molecular docking (ASN215, PRO261, CYS270, and TYR468) Interaction confirmed by thermal shift assay Decrease in TLR7 expression and activation of MyD88	[51]

## Data Availability

The original contributions presented in this study are included in the article. Further inquiries can be directed to the corresponding authors.

## References

[1] Cavaillon J.-M., Cavaillon J.-M., Singer M. (2017). Pathogen-Associated Molecular Patterns. Inflammation: From Molecular and Cellular Mechanisms to the Clinic.

[2] Chen R., Zou J., Liu J., Kang R., Tang D. (2025). DAMPs in the Immunogenicity of Cell Death. Mol. Cell.

[3] Kawai T., Ikegawa M., Ori D., Akira S. (2024). Decoding Toll-like Receptors: Recent Insights and Perspectives in Innate Immunity. Immunity.

[4] Sharma V., Sharma P., Singh T.G. (2024). Mechanistic Insights on TLR-4 Mediated Inflammatory Pathway in Neurodegenerative Diseases. Pharmacol. Rep..

[5] Pimentel-Nunes P., Soares J.B., Roncon-Albuquerque R., Dinis-Ribeiro M., Leite-Moreira A.F. (2010). Toll-like Receptors as Therapeutic Targets in Gastrointestinal Diseases. Expert Opin. Ther. Targets.

[6] Savva A., Roger T. (2013). Targeting Toll-like Receptors: Promising Therapeutic Strategies for the Management of Sepsis-Associated Pathology and Infectious Diseases. Front. Immunol..

[7] Molteni M., Bosi A., Rossetti C. (2018). Natural Products with Toll-Like Receptor 4 Antagonist Activity. Int. J. Inflamm..

[8] Gao W., Sun X., Li D., Sun L., He Y., Wei H., Jin F., Cao Y. (2020). Toll-like Receptor 4, Toll-like Receptor 7 and Toll-like Receptor 9 Agonists Enhance Immune Responses against Blood-Stage Plasmodium Chabaudi Infection in BALB/c Mice. Int. Immunopharmacol..

[9] Monserrat-Mesquida M., Quetglas-Llabrés M., Capó X., Bouzas C., Mateos D., Pons A., Tur J.A., Sureda A. (2020). Metabolic Syndrome Is Associated with Oxidative Stress and Proinflammatory State. Antioxidants.

[10] Muzio M., Mantovani A. (2000). Toll-like Receptors. Microbes Infect..

[11] Li C., Zheng X., Chang M., Tian Q., He Z., Tang Z., Chen X., Liu X., Yang D., Yan T. (2025). Toll-like Receptor-4 in the Fish Immune System. Dev. Comp. Immunol..

[12] Federico S., Pozzetti L., Papa A., Carullo G., Gemma S., Butini S., Campiani G., Relitti N. (2020). Modulation of the Innate Immune Response by Targeting Toll-like Receptors: A Perspective on Their Agonists and Antagonists. J. Med. Chem..

[13] Wang K., Huang H., Zhan Q., Ding H., Li Y. (2024). Toll-like Receptors in Health and Disease. MedComm.

[14] Warshakoon H.J., Hood J.D., Kimbrell M.R., Malladi S., Wu W.Y., Shukla N.M., Agnihotri G., Sil D., David S.A. (2009). Potential Adjuvantic Properties of Innate Immune Stimuli. Hum. Vaccines.

[15] Arancibia S.A., Beltrán C.J., Aguirre I.M., Silva P., Peralta A.L., Malinarich F., Hermoso M.A. (2007). Toll-like Receptors Are Key Participants in Innate Immune Responses. Biol. Res..

[16] Manavalan B., Basith S., Choi S. (2011). Similar Structures but Different Roles—An Updated Perspective on TLR Structures. Front. Physiol..

[17] Behzadi P., García-Perdomo H.A., Karpiński T.M. (2021). Toll-Like Receptors: General Molecular and Structural Biology. J. Immunol. Res..

[18] Neganova M.E., Yudaev P.A., Aleksandrova Y.R., Brel V.K. (2025). Monocarbonyl Synthetic Curcumin Analogues with Antitumour Potential. Chemical and Biological Aspects. Russ. Chem. Rev..

[19] Kumar H., Kawai T., Akira S. (2009). Toll-like Receptors and Innate Immunity. Biochem. Biophys. Res. Commun..

[20] Anders H.-J., Schlöndorff D. (2007). Toll-like Receptors: Emerging Concepts in Kidney Disease. Curr. Opin. Nephrol. Hypertens..

[21] Brennan J.J., Gilmore T.D. (2018). Evolutionary Origins of Toll-like Receptor Signaling. Mol. Biol. Evol..

[22] Mitchell J.A., Sorrentino R., Cartwright N., Paul-Clark M., Hansel T.T., Barnes P.J. (2010). Toll-like Receptor 2 as Therapeutic Target in Lung Disease. New Drugs and Targets for Asthma and COPD.

[23] Chun K.-H., Seong S.-Y. (2010). CD14 but Not MD2 Transmit Signals from DAMP. Int. Immunopharmacol..

[24] Cheng F., Yang F., Xiao S., Zhou Y., Wu Y., Zhang J., He Z. (2026). Targeting MD2 in Diseases: From Structure and Function to Inhibitor Development. Eur. J. Med. Chem..

[25] Chen L., Fu W., Zheng L., Wang Y., Liang G. (2018). Recent Progress in the Discovery of Myeloid Differentiation 2 (MD2) Modulators for Inflammatory Diseases. Drug Discov. Today.

[26] Mullen G.E.D., Kennedy M.N., Visintin A., Mazzoni A., Leifer C.A., Davies D.R., Segal D.M. (2003). The Role of Disulfide Bonds in the Assembly and Function of MD-2. Proc. Natl. Acad. Sci. USA.

[27] Batin Rahaman S.K., Nandi S.K., Mandal S.K., Debnath U. (2025). Structural Diversity and Mutational Challenges of Toll-Like Receptor 4 Antagonists as Inflammatory Pathway Blocker. Drug Dev. Res..

[28] Chen B., Di B. (2024). Endogenous Ligands of TLR4 in Microglia: Potential Targets for Related Neurological Diseases. Curr. Drug Targets.

[29] Negishi H., Endo N., Nakajima Y., Nishiyama T., Tabunoki Y., Nishio J., Koshiba R., Matsuda A., Matsuki K., Okamura T. (2019). Identification of U11snRNA as an Endogenous Agonist of TLR7-Mediated Immune Pathogenesis. Proc. Natl. Acad. Sci. USA.

[30] Martin D.B., Gaspari A.A., Gaspari A.A., Tyring S.K. (2008). Toll-like Receptors. Clinical and Basic Immunodermatology.

[31] Yin C.-Z., Li D., Cai J., Xu H.-L., Wang L.-J., Zheng M.-H., Liu H. (2022). Role of Toll-like receptor 7 in anti-infective immunity. Chin. J. Parasitol. Parasit. Dis..

[32] Cai X., Xu Y., Cheung A.K., Tomlinson R.C., Alcázar-Román A., Murphy L., Billich A., Zhang B., Feng Y., Klumpp M. (2013). PIKfyve, a Class III PI Kinase, Is the Target of the Small Molecular IL-12/IL-23 Inhibitor Apilimod and a Player in Toll-like Receptor Signaling. Chem. Biol..

[33] Yesudhas D., Gosu V., Anwar M.A., Choi S. (2014). Multiple Roles of Toll-like Receptor 4 in Colorectal Cancer. Front. Immunol..

[34] Moradi-Marjaneh R., Hassanian S.M., Fiuji H., Soleimanpour S., Ferns G.A., Avan A., Khazaei M. (2018). Toll like Receptor Signaling Pathway as a Potential Therapeutic Target in Colorectal Cancer. J. Cell. Physiol..

[35] Liu X., Zheng J., Zhou H. (2011). TLRs as Pharmacological Targets for Plant-Derived Compounds in Infectious and Inflammatory Diseases. Int. Immunopharmacol..

[36] Zhao Y., Wu J., Liu X., Chen X., Wang J. (2024). Decoding Nature: Multi-Target Anti-Inflammatory Mechanisms of Natural Products in the TLR4/NF-κB Pathway. Front. Pharmacol..

[37] Hariri M., Ghiasvand R. (2016). Cinnamon and Chronic Diseases. Advances in Experimental Medicine and Biology.

[38] Rao P.V., Gan S.H. (2014). Cinnamon: A Multifaceted Medicinal Plant. Evid.-Based Complement. Altern. Med..

[39] Pasupuleti V.R. (2021). Bioactive Compounds of Cinnamon (*Cinnamomum* Species). Reference Series in Phytochemistry.

[40] Pandey D.K., Chaudhary R., Dey A., Nandy S., Banik R.M., Malik T., Dwivedi P., Singh J., Meshram V., Gupta M. (2020). Current Knowledge of *Cinnamomum* Species: A Review on the Bioactive Components, Pharmacological Properties, Analytical and Biotechnological Studies. Bioactive Natural Products in Drug Discovery.

[41] Błaszczyk N., Rosiak A., Kałużna-Czaplińska J. (2021). The Potential Role of Cinnamon in Human Health. Forests.

[42] Senevirathne B.S., Jayasinghe M.A., Pavalakumar D., Siriwardhana C.G. (2022). Ceylon Cinnamon: A Versatile Ingredient for Futuristic Diabetes Management. J. Future Foods.

[43] Bai Z., Zhao H., Wang X., Wang Y., Chen Y., Chen G., Zhang Z., Zhao L. (2026). A Review on Factors Influencing the Chemical Composition and Functional Activity of Cinnamon Essential Oil. Food Sci..

[44] Muhammad D.R.A., Dewettinck K. (2017). Cinnamon and Its Derivatives as Potential Ingredient in Functional Food—A Review. Int. J. Food Prop..

[45] Katiyar A., Katiyar P., Sharma D. (2025). Therapeutic Uses of Cinnamon (*Cinnamomum verum*) in Human Health: A Review. Indian J. Agric. Biochem..

[46] Tamim A.A., Alharbi H., Hawsawi E., Aldosari Z., Alqahtani A. (2025). Optimized HPLC-DAD Method for Quantifying Coumarin and Cinnamaldehyde in Local Cinnamon Sample. Appl. Food Res..

[47] Samanth M., Bhat M. (2024). Synthesis and Importance of Coumarin Derivatives in Medicinal Chemistry: A Comprehensive Review. Russ. J. Bioorganic Chem..

[48] Diker N.Y., Kaplan O., Özen G.Ü., Çelebier M., Büyüktuncer Z., Çankaya İ.İ. (2026). Assessment of Coumarin Levels in Cinnamon Spices Available in the Turkish Market. Ank. Univ. Eczaci. Fak. Derg..

[49] Hong J.-W., Yang G.-E., Kim Y.B., Eom S.H., Lew J.-H., Kang H. (2012). Anti-Inflammatory Activity of Cinnamon Water Extract in Vivo and in Vitro LPS-Induced Models. BMC Complement. Altern. Med..

[50] Park T.G., Kim Y.R., Park S.-Y., Choi K., Kim K.J., Kim J.Y. (2023). Cinnamon (*Cinnamomum cassia*) Hot Water Extract Improves Inflammation and Tight Junctions in the Intestine In Vitro and In Vivo. Food Sci. Biotechnol..

[51] Maalouly G., Saliba Y., Hajal J., Zein-El-Din A., Fakhoury L., Najem R., Smayra V., Nassereddine H., Fares N. (2025). *Cinnamomum cassia* Alleviates Neuropsychiatric Lupus in a Murine Experimental Model. Nutrients.

[52] Shishehbor F., Rezaeyan Safar M., Rajaei E., Haghighizadeh M.H. (2018). Cinnamon Consumption Improves Clinical Symptoms and Inflammatory Markers in Women With Rheumatoid Arthritis. J. Am. Coll. Nutr..

[53] Liu F., Yang Y., Dong H., Zhu Y., Feng W., Wu H. (2024). Essential Oil from *Cinnamomum cassia* Presl Bark Regulates Macrophage Polarization and Ameliorates Lipopolysaccharide-Induced Acute Lung Injury through TLR4/MyD88/NF-κB Pathway. Phytomedicine.

[54] Zhao H., Zhang M., Zhou F., Cao W., Bi L., Xie Y., Yang Q., Wang S. (2016). Cinnamaldehyde Ameliorates LPS-Induced Cardiac Dysfunction via TLR4-NOX4 Pathway: The Regulation of Autophagy and ROS Production. J. Mol. Cell. Cardiol..

[55] Lee S.-C., Wang S.-Y., Li C.-C., Liu C.-T. (2018). Anti-Inflammatory Effect of Cinnamaldehyde and Linalool from the Leaf Essential Oil of *Cinnamomum osmophloeum* Kanehira in Endotoxin-Induced Mice. J. Food Drug Anal..

[56] Chung J., Kim S., Lee H.A., Park M.H., Kim S., Song Y.R., Na H.S. (2018). Trans-Cinnamic Aldehyde Inhibits Aggregatibacter Actinomycetemcomitans-Induced Inflammation in THP-1-Derived Macrophages via Autophagy Activation. J. Periodontol..

[57] Zareie A., Soleimani D., Askari G., Jamialahmadi T., Guest P.C., Bagherniya M., Sahebkar A. (2021). Cinnamon: A Promising Natural Product Against COVID-19. Advances in Experimental Medicine and Biology.

[58] Lucas K., Ackermann M., Leifke A.L., Li W.W., Pöschl U., Fröhlich-Nowoisky J. (2021). Ceylon Cinnamon and Its Major Compound Cinnamaldehyde Can Limit Overshooting Inflammatory Signaling and Angiogenesis In Vitro: Implications for COVID-19 Treatment. bioRxiv.

[59] Chen P., Zhou J., Ruan A., Zeng L., Liu J., Wang Q. (2022). Cinnamic Aldehyde, the Main Monomer Component of Cinnamon, Exhibits Anti-Inflammatory Property in OA Synovial Fibroblasts via TLR4/MyD88 Pathway. J. Cell. Mol. Med..

[60] Chen P., Ruan A., Zhou J., Huang L., Zhang X., Ma Y., Wang Q. (2020). Cinnamic Aldehyde Inhibits Lipopolysaccharide-Induced Chondrocyte Inflammation and Reduces Cartilage Degeneration by Blocking the Nuclear Factor-Kappa B Signaling Pathway. Front. Pharmacol..

[61] Li W., Wang K., Liu Y., Wu H., He Y., Li C., Wang Q., Su X., Yan S., Su W. (2022). A Novel Drug Combination of Mangiferin and Cinnamic Acid Alleviates Rheumatoid Arthritis by Inhibiting TLR4/NFκB/NLRP3 Activation-Induced Pyroptosis. Front. Immunol..

[62] Rezaei Z., Momtaz S., Gharazi P., Rahimifard M., Baeeri M., Abdollahi A.R., Abdollahi M., Niknejad A., Khayatan D., Farzaei M.H. (2024). Cinnamic Acid Ameliorates Acetic Acid-Induced Inflammatory Response through Inhibition of TLR-4 in Colitis Rat Model. Anti-Inflamm. Anti-Allergy Agents Med. Chem..

[63] Tan X., Wen Y., Han Z., Su X., Peng J., Chen F., Wang Y., Wang T., Wang C., Ma K. (2023). Cinnamaldehyde Ameliorates Dextran Sulfate Sodium-Induced Colitis in Mice by Modulating TLR4/NF-κB Signaling Pathway and NLRP3 Inflammasome Activation. Chem. Biodivers..

[64] Maalouly G., Itani T., Fares N. (2025). *Cinnamomum cassia* Modulates Key Players of Gut-Liver Axis in Murine Lupus. Biomedicines.

[65] Maalouly G., Martin C.-M.-A., Baz Y., Saliba Y., Baramili A.-M., Fares N. (2024). Antioxidant and Anti-Apoptotic Neuroprotective Effects of Cinnamon in Imiquimod-Induced Lupus. Antioxidants.

[66] Hajinejad M., Ghaddaripouri M., Dabzadeh M., Forouzanfar F., Sahab-Negah S. (2020). Natural Cinnamaldehyde and Its Derivatives Ameliorate Neuroinflammatory Pathways in Neurodegenerative Diseases. BioMed Res. Int..

[67] Zhao J., Zhang X., Dong L., Wen Y., Zheng X., Zhang C., Chen R., Zhang Y., Li Y., He T. (2015). Cinnamaldehyde Inhibits Inflammation and Brain Damage in a Mouse Model of Permanent Cerebral Ischaemia. Br. J. Pharmacol..

[68] Liu X., Lu Z., He G., Gao W., Hu K., Pan J., Zhao Y., Wang X., Yang G., Xu Y. (2025). Trans-Cinnamaldehyde Ameliorates Neuroinflammation-Mediated Synaptic Plasticity and Memory Impairment by Blocking TLR4/MyD88/MAPKs Pathway in Global Cerebral Ischemia. Brain Res. Bull..

[69] Zhong G., Shi J., Jiang Y., Li S., Wang X., Zhang T., Chen Z., Wang Q., Liu S. (2026). Mechanistic Study on the Alleviating Effects of Cinnamaldehyde on Aluminum Chloride-Induced Cognitive Impairment in Zebrafish through Modulating the TLR4/NF-κB Signaling Pathway. Metab. Brain Dis..

[70] Kang L.-L., Zhang D.-M., Ma C.-H., Zhang J.-H., Jia K.-K., Liu J.-H., Wang R., Kong L.-D. (2016). Cinnamaldehyde and Allopurinol Reduce Fructose-Induced Cardiac Inflammation and Fibrosis by Attenuating CD36-Mediated TLR4/6-IRAK4/1 Signaling to Suppress NLRP3 Inflammasome Activation. Sci. Rep..

[71] Şen H., Ertuğrul T. (2022). Investigation of the Effect of Cinnamon Extract on TLR4 Expression and Numerical Distribution of Mast Cells in the Experimental Diabetic Rat Kidney. Firat Univ. Saglik Bilim. Vet. Derg..

[72] Mei J., Ma J., Xu Y., Wang Y., Hu M., Ma F., Qin Z., Xue R., Tao N. (2020). Cinnamaldehyde Treatment of Prostate Cancer-Associated Fibroblasts Prevents Their Inhibitory Effect on T Cells Through Toll-Like Receptor 4. Drug Des. Dev. Ther..

[73] He W., Zhang W., Zheng Q., Wei Z., Wang Y., Hu M., Ma F., Tao N., Luo C. (2019). Cinnamaldehyde Causes Apoptosis of Myeloid-derived Suppressor Cells through the Activation of TLR4. Oncol. Lett..

[74] Momtaz S., Navabakhsh M., Bakouee N., Dehnamaki M., Rahimifard M., Baeeri M., Abdollahi A., Abdollahi M., Farzaei M.H., Abdolghaffari A.H. (2021). Cinnamaldehyde Targets TLR-4 and Inflammatory Mediators in Acetic-Acid Induced Ulcerative Colitis Model. Biologia.

[75] Peng J., Song X., Yu W., Pan Y., Zhang Y., Jian H., He B. (2024). The Role and Mechanism of Cinnamaldehyde in Cancer. J. Food Drug Anal..

[76] Shakir S.U., Khan S.A., Ullah M., Khan A., Khan N.R., Khan I. (2026). Recent Advancements and Clinical Translation of Cinnamaldehyde Derivatives in Therapeutics. Drug Dev. Ind. Pharm..

[77] Husain I., Gurley B.J., Kothapalli H.B., Wang Y.-H., Vedova L.D., Chittiboyina A.G., Khan I.A., Khan S.I. (2024). Evaluation of Bioaccessibility, Metabolic Clearance and Interaction with Xenobiotic Receptors (PXR and AhR) of Cinnamaldehyde. Food Chem. Mol. Sci..

[78] Chen Y., Ma Y., Ma W. (2009). Pharmacokinetics and Bioavailability of Cinnamic Acid after Oral Administration of Ramulus Cinnamomi in Rats. Eur. J. Drug Metab. Pharmacokinet..

[79] Zhao H., Xie Y., Yang Q., Cao Y., Tu H., Cao W., Wang S. (2014). Pharmacokinetic Study of Cinnamaldehyde in Rats by GC-MS after Oral and Intravenous Administration. J. Pharm. Biomed. Anal..

[80] Huang Y., Edirisinghe I., Burton-Freeman B.M., Sandhu A.K. (2023). Characterization and Pharmacokinetic Profile of Herbs and Spices’ Phytochemicals over 24 h after Consumption in Overweight/Obese Adults. Mol. Nutr. Food Res..

[81] Cheung C., Hotchkiss S.A.M., Pease C.K.S. (2003). Cinnamic Compound Metabolism in Human Skin and the Role Metabolism May Play in Determining Relative Sensitisation Potency. J. Dermatol. Sci..

[82] Zhu R., Liu H., Liu C., Wang L., Ma R., Chen B., Li L., Niu J., Fu M., Zhang D. (2017). Cinnamaldehyde in Diabetes: A Review of Pharmacology, Pharmacokinetics and Safety. Pharmacol. Res..

[83] Pigera S., Ranasinghe P., Kaumal M.N., Galappatthy P. (2023). Quantification of Metabolite Cinnamic Acid of Cinnamon (*Cinnamomum zeylanicum*) in Human Plasma. J. Natl. Sci. Found. Sri Lanka.

[84] Pahan K. (2011). Immunomodulation of Experimental Allergic Encephalomyelitis by Cinnamon Metabolite Sodium Benzoate. Immunopharmacol. Immunotoxicol..

[85] Abraham K., Pfister M., Wöhrlin F., Lampen A. (2011). Relative Bioavailability of Coumarin from Cinnamon and Cinnamon-Containing Foods Compared to Isolated Coumarin: A Four-Way Crossover Study in Human Volunteers. Mol. Nutr. Food Res..

[86] Tjandrawinata R.R., Rosari B.P., Syahputra R.A., Surya R., Nurkolis F. (2026). Cinnamon-Derived Phytonutrients as Modulators of Ion Channels and G Protein-Coupled Receptor Signaling in Metabolic Diseases. Nutrients.

[87] Ulbricht C., Seamon E., Windsor R.C., Armbruester N., Bryan J.K., Costa D., Giese N., Gruenwald J., Iovin R., Isaac R. (2011). An Evidence-Based Systematic Review of Cinnamon (*Cinnamomum* Spp.) by the Natural Standard Research Collaboration. J. Diet. Suppl..

[88] Asadi A., Kalalinia F., Marouzi S., Salmasi Z., Hashemi M. (2025). An Overview of the Therapeutic Applications of Cinnamon-Loaded in Nanosystems. Nanomed. J..

[89] Gajewska S., Klimowicz A., Wróblewska A. (2026). Comparative Study of Antioxidant Activity and Chemical Composition of Alcoholic Extracts and Essential Oils from *Cinnamomum verum* and *Cinnamomum cassia*. Food Chem..

[90] Huseinović E., Dedić J., Mehmedović E., Horozić E. (2025). Influence of Some Solvents and Solvent Mixtures on Polyphenol Content and Antioxidant Activity of *Cinnamomum verum* Extracts. Int. Res. J. Pure Appl. Chem..

[91] Chen X., Shang S., Yan F., Jiang H., Zhao G., Tian S., Chen R., Chen D., Dang Y. (2023). Antioxidant Activities of Essential Oils and Their Major Components in Scavenging Free Radicals, Inhibiting Lipid Oxidation and Reducing Cellular Oxidative Stress. Molecules.

[92] Gulcin I., Kaya R., Goren A.C., Akincioglu H., Topal M., Bingol Z., Çakmak K.C., Sarikaya S.B.O., Durmaz L., Alwasel S. (2019). Anticholinergic, Antidiabetic and Antioxidant Activities of Cinnamon (*Cinnamomum verum*) Bark Extracts: Polyphenol Contents Analysis by LC-MS/MS. Int. J. Food Prop..

[93] Sharifi-Rad J., Dey A., Koirala N., Shaheen S., El Omari N., Salehi B., Goloshvili T., Cirone Silva N.C., Bouyahya A., Vitalini S. (2021). *Cinnamomum* Species: Bridging Phytochemistry Knowledge, Pharmacological Properties and Toxicological Safety for Health Benefits. Front. Pharmacol..

[94] Roussel A.-M., Hininger I., Benaraba R., Ziegenfuss T.N., Anderson R.A. (2009). Antioxidant Effects of a Cinnamon Extract in People with Impaired Fasting Glucose That Are Overweight or Obese. J. Am. Coll. Nutr..

[95] Bastos M.S., Del Vesco A.P., Santana T.P., Santos T.S., de Oliveira Junior G.M., Fernandes R.P.M., Barbosa L.T., Gasparino E. (2017). The Role of Cinnamon as a Modulator of the Expression of Genes Related to Antioxidant Activity and Lipid Metabolism of Laying Quails. PLoS ONE.

[96] Sharma U.K., Sharma A.K., Pandey A.K. (2016). Medicinal Attributes of Major Phenylpropanoids Present in Cinnamon. BMC Complement. Altern. Med..

[97] Gülçin İ. (2011). Antioxidant Activity of Eugenol: A Structure–Activity Relationship Study. J. Med. Food.

[98] Wondrak G.T., Villeneuve N.F., Lamore S.D., Bause A.S., Jiang T., Zhang D.D. (2010). The Cinnamon-Derived Dietary Factor Cinnamic Aldehyde Activates the Nrf2-Dependent Antioxidant Response in Human Epithelial Colon Cells. Molecules.

[99] Agadzhanyan V.S., Oganesyan E.T., Abaev V.T. (2010). Targeted Search for a Lead Compound in a Series of Cinnamic Acid Derivatives Possessing Antiradical Activity. Pharm. Chem. J..

[100] Jiang H., Sun S.-L., Zhang C., Yuan E., Wei Q.-Y., Zeng Z. (2013). Antioxidative Activities of Natural Hydroxy-Bearing Cinnamaldehydes and Cinnamic Acids: A Comparative Study. Trop. J. Pharm. Res..

[101] Takao K., Toda K., Saito T., Sugita Y. (2017). Synthesis of Amide and Ester Derivatives of Cinnamic Acid and Its Analogs: Evaluation of Their Free Radical Scavenging and Monoamine Oxidase and Cholinesterase Inhibitory Activities. Chem. Pharm. Bull..

[102] González Moa M.J., Mandado M., Mosquera R.A. (2006). QTAIM Charge Density Study of Natural Cinnamic Acids. Chem. Phys. Lett..

[103] Da Silva Porto P.A.L., Laranjinha J.A.N., De Freitas V.A.P. (2003). Antioxidant Protection of Low Density Lipoprotein by Procyanidins: Structure/Activity Relationships. Biochem. Pharmacol..

[104] Li Z., Wang W., Cheng P., Dong Q., Chen H., Fu K., Fu C., Pu Y., Liu D. (2026). Proanthocyanidins in Food and Health: Structure–Activity Relationships, Application Challenges, and Emerging Strategies. Food Chem..

[105] Youn H.S., Lee J.K., Choi Y.J., Saitoh S.I., Miyake K., Hwang D.H., Lee J.Y. (2008). Cinnamaldehyde Suppresses Toll-like Receptor 4 Activation Mediated through the Inhibition of Receptor Oligomerization. Biochem. Pharmacol..

[106] Peri F., Calabrese V. (2014). Toll-like Receptor 4 (TLR4) Modulation by Synthetic and Natural Compounds: An Update. J. Med. Chem..

[107] Schink A., Naumoska K., Kitanovski Z., Kampf C.J., Fröhlich-Nowoisky J., Thines E., Pöschl U., Schuppan D., Lucas K. (2018). Anti-Inflammatory Effects of Cinnamon Extract and Identification of Active Compounds Influencing the TLR2 and TLR4 Signaling Pathways. Food Funct..

[108] Ho S.-C., Chang Y.-H., Chang K.-S. (2018). Structural Moieties Required for Cinnamaldehyde-Related Compounds to Inhibit Canonical IL-1β Secretion. Molecules.

[109] Meng J., Lien E., Golenbock D.T. (2010). MD-2-Mediated Ionic Interactions between Lipid A and TLR4 Are Essential for Receptor Activation. J. Biol. Chem..

[110] Slivka P.F., Shridhar M., Lee G., Sammond D.W., Hutchinson M.R., Martinko A.J., Buchanan M.M., Sholar P.W., Kearney J.J., Harrison J.A. (2009). A Peptide Antagonist of the TLR4–MD2 Interaction. Chembiochem.

[111] Zhao L., Lee J.Y., Hwang D.H. (2011). Inhibition of Pattern Recognition Receptor-Mediated Inflammation by Bioactive Phytochemicals: A Review of Recent Research. Nutr. Rev..

[112] Wang S., Song X., Zhang K., Deng S., Jiao P., Qi M., Lian Z., Yao Y. (2020). Overexpression of Toll-Like Receptor 4 Affects Autophagy, Oxidative Stress, and Inflammatory Responses in Monocytes of Transgenic Sheep. Front. Cell Dev. Biol..

[113] Davoudi F., Ramazani E. (2024). Antioxidant and Anti-Inflammatory Effects of *Cinnamomum* Species and Their Bioactive Compounds: An Updated Review of the Molecular Mechanisms. Physiol. Pharmacol..

[114] Asehnoune K., Strassheim D., Mitra S., Kim J.Y., Abraham E. (2004). Involvement of Reactive Oxygen Species in Toll-Like Receptor 4-Dependent Activation of NF-κB. J. Immunol..

[115] Cong W., Ruan D., Xuan Y., Niu C., Tao Y., Wang Y., Zhan K., Cai L., Jin L., Tan Y. (2015). Cardiac-Specific Overexpression of Catalase Prevents Diabetes-Induced Pathological Changes by Inhibiting NF-κB Signaling Activation in the Heart. J. Mol. Cell. Cardiol..

[116] Kim J.-H., Na H.-J., Kim C.-K., Kim J.-Y., Ha K.-S., Lee H., Chung H.-T., Kwon H.J., Kwon Y.-G., Kim Y.-M. (2008). The Non-Provitamin A Carotenoid, Lutein, Inhibits NF-κB-Dependent Gene Expression through Redox-Based Regulation of the Phosphatidylinositol 3-Kinase/PTEN/Akt and NF-κB-Inducing Kinase Pathways: Role of H_2_O_2_ in NF-κB Activation. Free Radic. Biol. Med..

[117] Su Q., Yu X.-J., Wang X.-M., Li H.-B., Li Y., Bai J., Qi J., Zhang N., Liu K.-L., Zhang Y. (2021). Bilateral Paraventricular Nucleus Upregulation of Extracellular Superoxide Dismutase Decreases Blood Pressure by Regulation of the NLRP3 and Neurotransmitters in Salt-Induced Hypertensive Rats. Front. Pharmacol..

[118] Kadhim H.M. (2016). Antiinflmmatory and Antihyperlipidemic Effect of Adjuvant Cinnamon in Type 2 Diabetic Patients. Int. J. Pharm. Sci. Rev. Res..

[119] Oh E.S., Petersen K.S., Kris-Etherton P.M., Rogers C.J. (2022). Four Weeks of Spice Consumption Lowers Plasma Proinflammatory Cytokines and Alters the Function of Monocytes in Adults at Risk of Cardiometabolic Disease: Secondary Outcome Analysis in a 3-Period, Randomized, Crossover, Controlled Feeding Trial. Am. J. Clin. Nutr..

[120] Zhang K., Li Y., Lin X., Daneshar M., Karamian F., Li M. (2024). Effect of Cinnamon Supplementation on Blood Pressure, Oxidative Stress, and Inflammatory Biomarkers in Adults: An Umbrella Review of the Meta-Analyses of Randomized Controlled Trials. Nutr. Metab. Cardiovasc. Dis..

[121] Wang F., Pu C., Zhou P., Wang P., Liang D., Wang Q., Hu Y., Li B., Hao X. (2015). Cinnamaldehyde Prevents Endothelial Dysfunction Induced by High Glucose by Activating Nrf2. Cell. Physiol. Biochem..

[122] Londono M.B.Z., Yunda D.F.U., Osma J.A., Maldonado-Celis M.E. (2022). Coffee, Ginger and Cinnamon: Molecular Mechanisms of Action on Human Health. Molecular Mechanisms of Functional Food.

[123] Yan J., Li J., Zhang L., Sun Y., Jiang J., Huang Y., Xu H., Jiang H., Hu R. (2018). Nrf2 Protects against Acute Lung Injury and Inflammation by Modulating TLR4 and Akt Signaling. Free Radic. Biol. Med..

[124] Huang J., Yue S., Ke B., Zhu J., Shen X.-D., Zhai Y., Yamamoto M., Busuttil R.W., Kupiec-Weglinski J.W. (2014). Nuclear Factor Erythroid 2yrelated Factor 2 Regulates Toll-like Receptor 4 Innate Responses in Mouse Liver Ischemia-Reperfusion Injury through Akt-Forkhead Box Protein O1 Signaling Network. Transplantation.

[125] Kwon H.-K., Hwang J.-S., Lee C.-G., So J.-S., Sahoo A., Im C.-R., Jeon W.K., Ko B.S., Lee S.H., Park Z.Y. (2011). Cinnamon Extract Suppresses Experimental Colitis through Modulation of Antigen-Presenting Cells. World J. Gastroenterol..

[126] Mondal S., Pahan K. (2015). Cinnamon Ameliorates Experimental Allergic Encephalomyelitis in Mice via Regulatory T Cells: Implications for Multiple Sclerosis Therapy. PLoS ONE.

[127] Chen L., Yang Y., Yuan P., Yang Y., Chen K., Jia Q., Li Y. (2014). Immunosuppressive Effects of A-Type Procyanidin Oligomers from *Cinnamomum tamala*. Evid. Based Complement. Altern. Med..

[128] Fukata E., Rohman M.S., Fakurazi S., Nik Malek N.A.N., Kawamoto Y., Endharti A.T. (2025). In Silico Prediction of Cinnamaldehyde on the PI3K/AKT Pathway Activator of Anti-Apoptotic Potential. J. Pharm. Pharmacogn. Res..

[129] Qureshi K.A., Mohammed S.A.A., Khan O., Ali H.M., El-Readi M.Z., Mohammed H.A. (2022). Cinnamaldehyde-Based Self-Nanoemulsion (CA-SNEDDS) Accelerates Wound Healing and Exerts Antimicrobial, Antioxidant, and Anti-Inflammatory Effects in Rats’ Skin Burn Model. Molecules.

[130] Korbecki J., Maruszewska A., Bosiacki M., Chlubek D., Baranowska-Bosiacka I. (2022). The Potential Importance of CXCL1 in the Physiological State and in Noncancer Diseases of the Cardiovascular System, Respiratory System and Skin. Int. J. Mol. Sci..

[131] Razagh Shazr N., Fathi M., Zarrinkavyani K., Biranvand Z. (2026). Effects of Cinnamon and Silver Nanoparticles Combination on Growth, Immune Function, Oxidative Status, Inflammation, and Cecal Microbiota in Broiler Chickens. J. Appl. Anim. Res..

[132] Kim A.-S., Chae C.-H., Kim J., Choi J.-Y., Kim S.-G., Băciut G. (2012). Silver Nanoparticles Induce Apoptosis through the Toll-like Receptor 2 Pathway. Oral Surg. Oral Med. Oral Pathol. Oral Radiol..

[133] Ibrahim E.A., Moawed F.S.M., Moustafa E.M. (2020). Suppression of Inflammatory Cascades via Novel Cinnamic Acid Nanoparticles in Acute Hepatitis Rat Model. Arch. Biochem. Biophys..

[134] Chen P., Xu Z., Wang X., He J., Yang J., Wang J., Chattipakorn N., Wu D., Tang Q., Liang G. (2023). Discovery of New Cinnamic Derivatives as Anti-Inflammatory Agents for Treating Acute Lung Injury in Mice. Arch. Pharm..

[135] Ka S.-M., Kuoping Chao L., Lin J.-C., Chen S.-T., Li W.-T., Lin C.-N., Cheng J.-C., Jheng H.-L., Chen A., Hua K.-F. (2016). A Low Toxicity Synthetic Cinnamaldehyde Derivative Ameliorates Renal Inflammation in Mice by Inhibiting NLRP3 Inflammasome and Its Related Signaling Pathways. Free Radic. Biol. Med..

[136] Reale M., Costantini E., Aielli L., Rienzo A.D., Biase G.D., Stefano A.D., Cacciatore I. (2024). Exploring the Therapeutic Potential of Cinnamoyl Derivatives in Attenuating Inflammation in Lipopolysaccharide-Induced Caco-2 Cells. Future Med. Chem..

[137] Zhang Y., Cao W., Xie Y.-H., Yang Q., Li X.-Q., Liu X.-X., Wang S.-W. (2012). The Comparison of α-Bromo-4-Chlorocinnamaldehyde and Cinnamaldehyde on Coxsackie Virus B3-Induced Myocarditis and Their Mechanisms. Int. Immunopharmacol..

[138] Pham L., Jiang R., Liu Z., Nguyen M., Nguyen Y., Gong Y., Bi Y., Kim H.-R., Kim Y.R., Kim G. (2023). Synthesis of 9-Cinnamyl-9H-Purine Derivatives as Novel TLR4/MyD88/NF-κB Pathway Inhibitors for Anti-Inflammatory Effects. ACS Med. Chem. Lett..

[139] Chen G., Zhang Y., Liu X., Fang Q., Wang Z., Fu L., Liu Z., Wang Y., Zhao Y., Li X. (2016). Discovery of a New Inhibitor of Myeloid Differentiation 2 from Cinnamamide Derivatives with Anti-Inflammatory Activity in Sepsis and Acute Lung Injury. J. Med. Chem..

[140] Al Nasr I.S., Koko W.S., Khan T.A., Schobert R., Biersack B. (2024). Antiparasitic Activities of Acyl Hydrazones from Cinnamaldehydes and Structurally Related Fragrances. Antibiotics.

[141] Karimirad R., Inbaraj B.S., Chen B.-H. (2025). Recent Advances on the Analysis and Biological Functions of Cinnamaldehyde and Its Derivatives. Antioxidants.

[142] Chun Y.L., Park K.-H., Pallavi B., Eom W.-J., Park C., Huh Y., Lee Y., Lee J., Kim S.H., Yeo S.G. (2022). Novel Cinnamaldehyde Derivatives Inhibit Peripheral Nerve Degeneration by Targeting Schwann Cells. Antioxidants.

